# Age-Related Transcriptional Deregulation of Genes Coding Synaptic Proteins in Alzheimer's Disease Murine Model: Potential Neuroprotective Effect of Fingolimod

**DOI:** 10.3389/fnmol.2021.660104

**Published:** 2021-07-09

**Authors:** Henryk Jęśko, Iga Wieczorek, Przemysław Leonard Wencel, Magdalena Gąssowska-Dobrowolska, Walter J. Lukiw, Robert Piotr Strosznajder

**Affiliations:** ^1^Department of Cellular Signalling, Mossakowski Medical Research Institute, Polish Academy of Sciences, Warsaw, Poland; ^2^Laboratory of Preclinical Research and Environmental Agents, Mossakowski Medical Research Institute, Polish Academy of Sciences, Warsaw, Poland; ^3^LSU Neuroscience Center, Departments of Neurology and Ophthalmology, Louisiana State University School of Medicine, New Orleans, LA, United States

**Keywords:** aging, Alzheimer's disease, amyloid β, FTY720/fingolimod, neurodegeneration, sphingolipids, sphingosine-1-phosphate, synaptic proteins

## Abstract

Alzheimer's disease (AD) induces time-dependent changes in sphingolipid metabolism, which may affect transcription regulation and neuronal phenotype. We, therefore, analyzed the influence of age, amyloid β precursor protein (AβPP), and the clinically approved, bioavailable sphingosine-1-phosphate receptor modulator fingolimod (FTY720) on the expression of synaptic proteins. RNA was isolated, reverse-transcribed, and subjected to real-time PCR. Expression of mutant (V717I) AβPP led to few changes at 3 months of age but reduced multiple mRNA coding for synaptic proteins in a 12-month-old mouse brain. Complexin 1 (*Cplx1*), SNAP25 (*Snap25*), syntaxin 1A (*Stx1a*), neurexin 1 (*Nrxn1*), neurofilament light (*Nefl*), and synaptotagmin 1 (*Syt1*) in the hippocampus, and VAMP1 (*Vamp1*) and neurexin 1 (*Nrxn1*) in the cortex were all significantly reduced in 12-month-old mice. Post mortem AD samples from the human hippocampus and cortex displayed lower expression of VAMP, synapsin, neurofilament light (NF-L) and synaptophysin. The potentially neuroprotective FTY720 reversed most AβPP-induced changes in gene expression (*Cplx1, Stx1a, Snap25*, and *Nrxn1*) in the 12-month-old hippocampus, which is thought to be most sensitive to early neurotoxic insults, but it only restored *Vamp1* in the cortex and had no influence in 3-month-old brains. Further study may reveal the potential usefulness of FTY720 in the modulation of deregulated neuronal phenotype in AD brains.

## Introduction

Aging creates a vulnerable background for the development of incurable neurodegenerative disorders, such as Alzheimer's disease (AD), which is characterized by the presence of extracellular senile plaques of amyloid β (Aβ) and neurofibrillary tangles of hyperphosphorylated tau protein. AD is the most common neurodegenerative disorder in the elderly. Its most frequent late-onset, usually sporadic form, follows a long period of stealthy, relatively symptom-free development. Major neuronal populations are already lost when first easily recognizable behavioral outcomes appear, which dramatically hampers both therapy and research on its etiology. Although AD is relatively less frequently caused by inherited genetic mutations, this form of disease raises hopes for a better understanding of AD mechanisms. Aggregation of Aβ peptides is one of the hallmarks of all AD forms, and overexpression of the Aβ precursor protein (AβPP) is frequently used to generate animal models of the disease. In pathological conditions, the normally dominating AβPP cleavage by α- and γ-secretase is partially replaced by amyloidogenic proteolysis by β- and γ-secretase. AβPP mutations may be responsible for the shift in AβPP processing and for the increased Aβ tendency to aggregate. Increasing the local concentration of Aβ, along with its ongoing aggregation, seems to be an important stimulus changing its activity from the supposed physiological stimulation of synaptic plasticity into a neurotoxin (Fagiani et al., 2019).

Although the precise location of Aβ in the chain of events leading to neurodegeneration is still debated, the presence of its excessive amounts in the extracellular space leads to signaling anomalies and free radical stress. Synaptic disturbances are part of early AD, preceding neuronal death by a significant time margin (Fagiani et al., 2019). While large extracellular deposits of Aβ do not correlate precisely with cognitive decline, oligomers and intracellular soluble/aggregated Aβ associate with ultrastructural damage to synapses/distal neurites, and with disease severity (Rajmohan and Reddy, 2017; Marsh and Alifragis, 2018). Oligomers are, therefore, currently viewed as the likely Aβ species capable of driving synaptic pathology (Walsh et al., 2002), which accompanies and most likely predates extensive neurodegeneration (Zamponi and Pigino, 2019). Synaptic alterations are targeted by few treatments currently available for a transient slowing of AD symptoms (Marsh and Alifragis, 2018). The molecular interactions that mediate the detrimental influence of Aβ on synapses most likely involve members and binding partners of the SNARE (soluble N-ethylmaleimide sensitive fusion attachment protein receptor) complex, which ensure structural integrity and regulate synaptic vesicle turnover (Russell et al., 2012; Yang et al., 2015; Koppensteiner et al., 2016; Marsh and Alifragis, 2018). Low concentrations of monomeric Aβ have been suggested to stimulate neurotransmission through stimulation of vesicle fusion (during neurotransmitter secretion) and inhibition of endocytosis (which mediates neurotransmitter removal from the synaptic cleft); pathological, aggregating Aβ, in turn, would predominantly block exocytosis (Fagiani et al., 2019). Aβ has also been found to regulate receptor activities and their feedback endocytosis (Kamenetz et al., 2003; Hsieh et al., 2006). Correspondingly, accumulating evidence suggests that elusive functions of Aβ in a healthy brain could include synaptic regulation (Gulisano et al., 2019). However, while protein–protein interactions of Aβ are gaining attention, comparatively little is known on the possible influence of Aβ/AβPP on the expression of genes, such as those that code synaptic proteins. Aβ interacts with synaptic translation machinery (Ghosh et al., 2020), but there is also a possibility that it could modulate gene expression through modification of the sphingolipid-dependent modulation of transcription factors (Jeśko et al., 2019a,b).

Altered signaling pathways are a major part of the known pathomechanism of synapse and neuron loss. Disturbances in bioactive sphingolipids are observed both in aging and AD (Katsel et al., 2007; Han, 2010; Couttas et al., 2014). Interestingly, these disturbances occur very early in the disease course and seem to diminish in later stages (Han, 2010). Ceramide and its derivative sphingosine typically activate apoptosis, while their respective phosphates (C1P: ceramide-1-phosphate; S1P: sphingosine-1-phosphate) are mostly positive regulators of cellular survival. S1P has a wide array of other functions, modulating acquisition and maintenance of neuronal phenotypes, such as neurotransmission and synaptic plasticity. S1P (and probably C1P) acts either through cell surface receptors, or as second messengers within the cell. The cell membrane receptors for S1P (S1PR1−5) bind to G_12_/_13_, G_q_, and G_i_ proteins, and are able to influence transcription factors, such as AP-1, NF-kB, p53, or the splicing regulator SRSF1 (Kaneider et al., 2004; Van Brocklyn and Williams, 2012; Patwardhan et al., 2014; Czubowicz et al., 2019; Jeśko et al., 2019a). Strikingly, Aβ is able to modify the expression of sphingolipid-related genes (Kaneider et al., 2004; Jeśko et al., 2019b, 2020). This may explain the above-mentioned shift from S1P signaling toward ceramide production, which is observed in the brain in the early stages of AD and preceding mild cognitive impairment (MCI) (Katsel et al., 2007; Han, 2010; Couttas et al., 2014). Increased ceramide production also occurs before MCI onset, leading to the idea of *pre-MCI*, a period when upstream events set the neurodegenerative processes in motion, likely with the significant engagement of sphingolipid-based pathways (Han, 2010). Besides altered survival/death signaling, changes in ceramide levels might also impact AβPP maturation and proteolytic processing, which can lead to increased generation of Aβ, closing the feedback circle of events (Puglielli et al., 2003; Sawamura et al., 2004; Tamboli et al., 2005). S1P has also been known to regulate various secretory pathways (Riganti et al., 2016). Somewhat scattered data also suggest the engagement of sphingolipids in the creation and maintenance of neuronal projections and synapses, which are among the earliest targets of Aβ neurotoxicity (Scheff et al., 2006; Ferreira et al., 2015).

The aim of the study was to characterize the influence of the expression of AβPP carrying familial AD–linked mutation (V717I) on the expression of genes coding selected synaptic proteins in the mouse brain cortex and hippocampus at the age of 3 and 12 months and to examine the impact of treatment with FTY720 (fingolimod, an S1P receptor modulator) on these changes. The results were verified with alterations observed in neocortical and hippocampal samples of human AD cases.

## Materials and Methods

Three- and 12-month-old FVB-Tg (Thy1; APP LD2/B6) mice that express AβPP harboring V717I “London” mutation predominantly in the brain and spinal cord neurons were used. The “London” mutation associates with early AD and increases the amounts of Aβ, changing the isoform ratios in favor of the highly neurotoxic Aβ_42_ (Jeśko et al., 2019b).

### Animals

Female FVB-Tg (Thy1; APP LD2/B6) mice overexpressed human AβPP with V717I (“London”) mutation under the control of a fragment of thy 1 promoter that ensured expression specifically in the neurons of the brain and spinal cord. The Animal House of the Mossakowski Medical Research Institute PAS, Warsaw, Poland maintained the mice under specific pathogen-free (SPF) conditions, under controlled temperature and humidity conditions, and a 12-h light/dark cycle. Animals aged 3 or 12 months were treated for 2 weeks daily (15 i.p. injections) with 1 mg/kg b.w. FTY720 (Cayman Chemical, Ann Arbor, Michigan, United States, cat. No 10006292) in 0.9% NaCl, or NaCl only (treatment controls), based on a previous study (Poti et al., 2012; Asle-Rousta et al., 2013; Jeśko et al., 2019b). Mice that did not inherit the transgene were used as transgene controls.

All possible measures were used to reduce the number of used animals and minimize their pain/distress. Initial experiments were performed on a minimal number of animals (typically *n* = 3–4), and only selected results were confirmed on larger cohorts. The experiments were approved by the IV Local Ethics Committee for Animal Experimentation in Warsaw and the Ministry of Environment (approval no. 67/2015 from July 2, 2016 and no. 139 from August 22, 2016, respectively) and were carried out in accordance with the EC Council Directive of November 24, 1986 (86/609/EEC) following the ARRIVE guidelines, the NIH Guide for the Care and Use of Laboratory Animals, and the “Guidelines for the Use of Animals in Neuroscience Research” by the Society for Neuroscience.

### Gene Expression Measurement in Mouse Brain Parts by Real-Time Polymerase Chain Reaction

A day after the last treatment, the animals were decapitated, and cerebral cortices and hippocampi were isolated on ice and flash-frozen in liquid nitrogen. RNA was extracted using TRI-reagent according to the protocol of the manufacturer (Sigma-Aldrich/Merck) and DNA digested with DNase I (Sigma-Aldrich, St. Louis, MO, USA). RNA quantity and quality were measured spectrophotometrically (A_260_/A_280_ method). Reverse transcription of 4 μg of total RNA was performed with avian myeloblastosis virus (AMV) enzyme and random sequence primers (High Capacity Reverse Transcription Kit, Applied Biosystems, Foster City, CA, United States). TaqMan Gene Expression Assay kits were used for real-time PCR on Applied Biosystems 7500 Real-Time PCR System (Applied Biosystems/Thermo Fisher Scientific, Foster City, California, USA, cat. No. 4331182). Mm01198853_m1 (*Cplx1*), Mm01315666_m1 (*Nefl*), Mm00456201_m1 (*Nefm*), Mm00660298_m1 (*Nrxn1*), Mm01276449_m1 (*Snap25*), Mm00444008_m1 (*Stx1a*), Mm00449772_m1 (*Syn1*), Mm00436850_m1 (*Syp*), Mm00436858_m1 (*Syt1*), Mm01185107_g1 (*Vamp1*). Gene expression in tri- to quadruplicate samples was calculated using the ddCt method and normalized against actin beta (Actb - Mm00607939_s1). All measurement plates for each brain part/age combination were calibrated with the same sample. Statistical significance was analyzed with a two-way analysis of variance (ANOVA; GraphPad Software, San Diego, CA, United States); “*p*” value < 0.05 was deemed statistically significant; experimental results are expressed as means ± SEM (standard error of the mean).

### Gene Expression Analysis of Human Post Mortem Brain Samples by Northern Dot Blot Arrays

A guanidine isothiocyanate- and silica gel-based membrane total RNA purification system and miRNA isolation kit (PureLink™ Invitrogen, Carlsbad, CA, United States) were used to isolate total RNA; total RNA concentrations were quantified using RNA 6000 Nano LabChips and a 2100 Bioanalyzer (Caliper Technologies, Mountainview, CA, United States; Agilent Technologies, Palo Alto, CA, United States). Synaptic and cytoskeletal RNA abundances were analyzed and quantified using Northern dot blot arrays as previously described (McLachlan et al., 1988; Lukiw et al., 1990, 2008, 2020). Altered RNA levels of interest were further verified using a quantitative Northern dot blot focusing assay that utilizes a T4 PNK kinase radiolabel system employing [α-^32^P]-dATP (6,000 Ci/m mol; Invitrogen, Carlsbad, CA, United States) that significantly interrogates the abundance of RNA and miRNA signals (Lukiw et al., 2008, 2020). Northern dot blot signal strengths were quantified using data-acquisition software provided with a GS250 molecular imager (Bio-Rad, Hercules, CA, United States), and graphic presentations (including comparative bar graphs) were performed using Excel algorithms (Microsoft, Seattle, WA, United States) and Adobe Photoshop 6.0 (Adobe Systems, San Jose, CA, United States). Alternately Northern dot blot patterns were analyzed using a cut-and count method. Statistical significance was analyzed using a two-way factorial analysis of variance (*p*, ANOVA; SAS Institute, Cary, NC, United States). A “*p*” value < 0.05 was deemed statistically significant; experimental values in the Figures are expressed as means ± standard deviation (SD) of that mean.

The acquisition, handling, experimental, and analytical procedures involving post mortem human brain tissues were carried out in an ethical manner in strict accordance with the ethics review board policies at brain and tissue donor institutions and at the Louisiana State University (LSU) Health Sciences Center. Informed consent from next of kin was obtained at brain and tissue donor institutions for all tissue samples prior to autopsy and donation; coded post mortem brain tissue samples (containing no personal identifying information of the donors) were obtained from the brain and tissue banks listed above. The ethical use of post mortem human brain tissues and their analyses were also carried out in strict accordance with the Institutional Biosafety Committee and the Institutional Review Board Committee (IBC/IRBC) ethical guidelines IBC#18059 and IRBC#6774 at the LSU Health Sciences Center, New Orleans, LA, 70112 United States. Project identification codes: NIA AG18031 and NIA AG038834.

## Results

To analyze the effects of Aβ precursor protein (AβPP) expression and administration of the potentially neuroprotective drug fingolimod (FTY720, Gilenya™) on the expression of genes coding for synaptic proteins, we used a mouse model (Jeśko et al., 2019b) expressing AβPP with V717I “London” mutation. The mutation is linked to familial FAD/early onset AD and stimulates the production of Aβ, especially its most toxic 42 amino acid species. Despite obvious limitations shared with other animal models of AD, the mice display a temporal sequence of behavioral alterations characteristic for AD and relatively closely follow disturbances of sphingolipid metabolism we noted in the human brain (Moechars et al., 1999; Van Dorpe et al., 2000; Jeśko et al., 2019b, 2020).

FTY720 is an S1P receptor modulator with characterized bioavailability, currently used because of its capacity to cause internalization of the receptors in immune cells in relapsing-remitting multiple sclerosis, primarily lymphocytes but also in brain astrocytes (Choi et al., 2011). The immune component may also be important for the action of FTY720 in other neurodegeneration-linked diseases (Becker-Krail et al., 2017). FTY720 also exerts its neuroprotective potential through enhanced production of brain-derived neurotrophic factor and modulation of its downstream signaling (Doi et al., 2013; Fukumoto et al., 2014; Becker-Krail et al., 2017). However, its primary recognized biological role is to activate S1PRs, potentially leading to a strong anti-apoptotic signal. Finally, FTY720 has been shown to positively modulate synaptic signaling and to restore long-term synaptic plasticity affected by neurodegenerative insults (Nazari et al., 2016; Darios et al., 2017; Zhang et al., 2020). As Aβ is capable of altering sphingolipid (especially S1P) metabolism, it might result in FTY720-treatable disturbances in survival/death pathways (Jeśko et al., 2019b). However, it might also deregulate the known engagement of sphingolipids in gene regulation, such as those coding for synaptic proteins (Cai et al., 2008).

**Figure 1:**
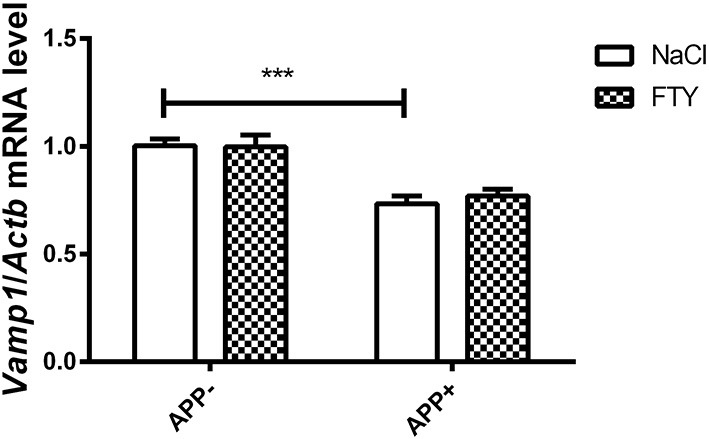
Levels of mRNA coding for synaptic protein VAMP1 in 3-month-old mouse cortex–effect of AβPP expression and FTY720 administration. Levels of mRNAs measured with real-time PCR in the cerebral cortex of 3-month-old (adult) mice as described in Materials and methods. Results from AβPP-expressing mice were compared with those from control animals that did not inherit the transgene. Effect of FTY720 administration was assessed against vehicle-treated animals of the same group. *Vamp1*, vesicle-associated membrane protein 1; APP^−^, animals without V717I AβPP transgene; APP^+^, mice expressing V717I AβPP transgene. ^***^p < 0.001 vs. corresponding controls, two-way ANOVA followed by Tukey's *post-hoc* test (significances marked over the horizontal bar describe the difference between vehicle-treated control animals and vehicle-treated AβPP-expressing mice). *n* = 6–7, measured in tri- to quadruplicate ± SEM.

Results from this study show reduced *Vamp1* mRNA in the cerebral cortex of 3-month-old mice expressing V717I AβPP transgene. Administration of FTY720 had no effect (Figure 1). *Cplx1, Nrxn1, Syn1*, and *Snap25* remained unchanged in the 3-month-old AβPP mouse brain cortex. Similarly, *Nefl* and *Nefm* were not changed (Supplementary Figure 1).

**Figure 2:**
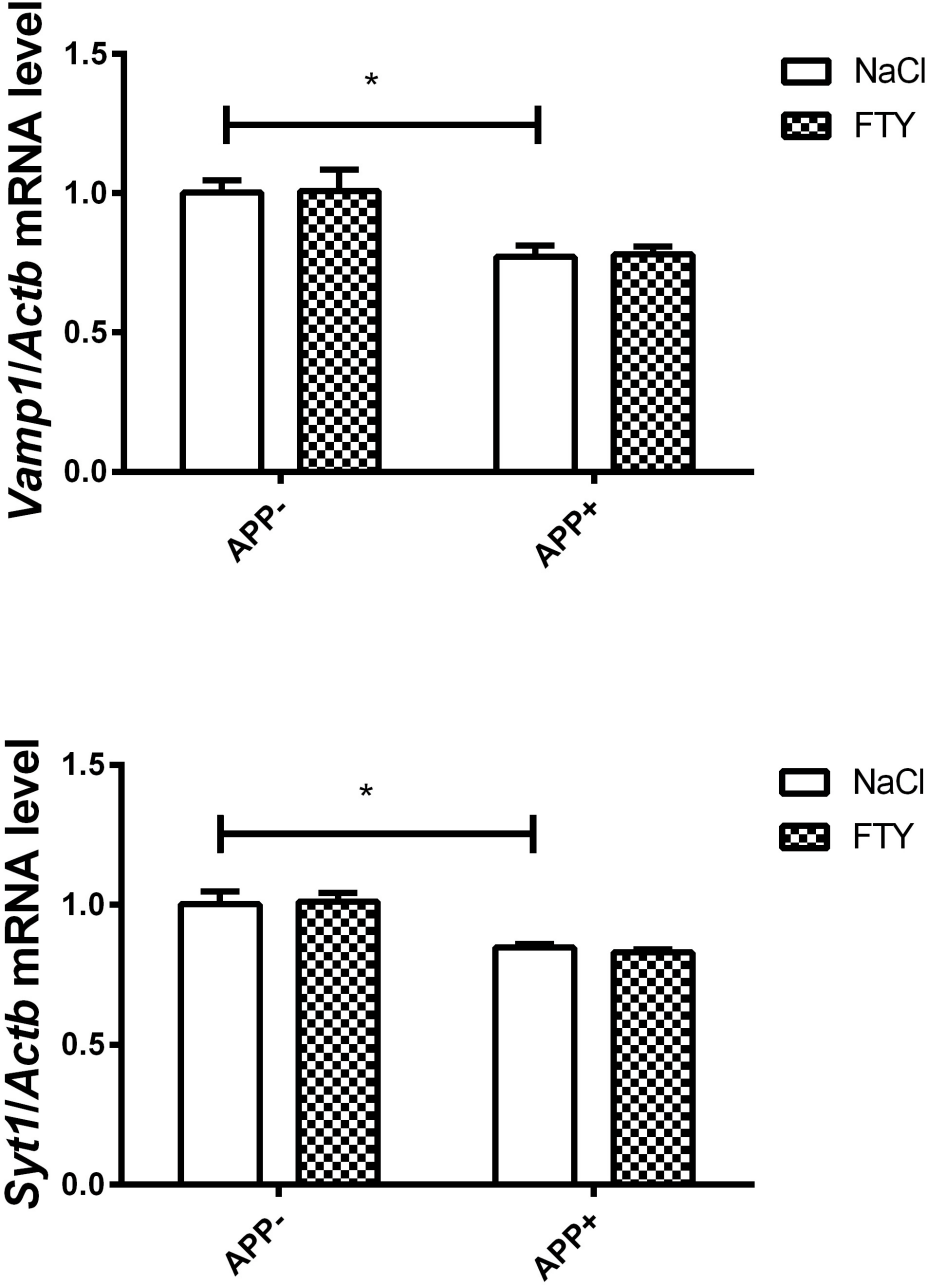
Levels of mRNAs coding for synaptic proteins VAMP1 and SYT1 in the 3-month-old mouse hippocampus: effect of AβPP and FTY720. Levels of mRNAs measured in the hippocampus of 3-month-old (adult) mice as described in Materials and methods. mRNA levels in AβPP-expressing mice were compared with control animals that did not inherit the transgene. Effect of FTY720 administration was assessed against vehicle-treated animals in each group. *Vamp1*, vesicle-associated membrane protein 1; *Syt1*, synaptotagmin 1; APP^−^, animals without V717I AβPP transgene; APP^+^, mice expressing V717I AβPP transgene. ^*^p < 0.05 vs. corresponding controls, two-way ANOVA followed by Tukey's *post-hoc* test (horizontal bars: effect of AβPP). *n* = 3–4, measured in triplicate ± SEM.

The pattern of mRNA changes in the hippocampus of 3-month-old AβPP-expressing mice also included a reduction in *Vamp1*; the SNARE-interacting partner *Syt1* was also significantly reduced. FTY720 had no effect on their levels (Figure 2). Among the SNARE proteins, we did not observe any changes in the expression of *Stx1a* or *Snap25* mRNA. *Syn1* showed some tendency toward reduction but did not reach significance (Supplementary Figure 2). *Nefl* and *Nefm* remained unchanged (Supplementary Figure 2).

**Figure 3:**
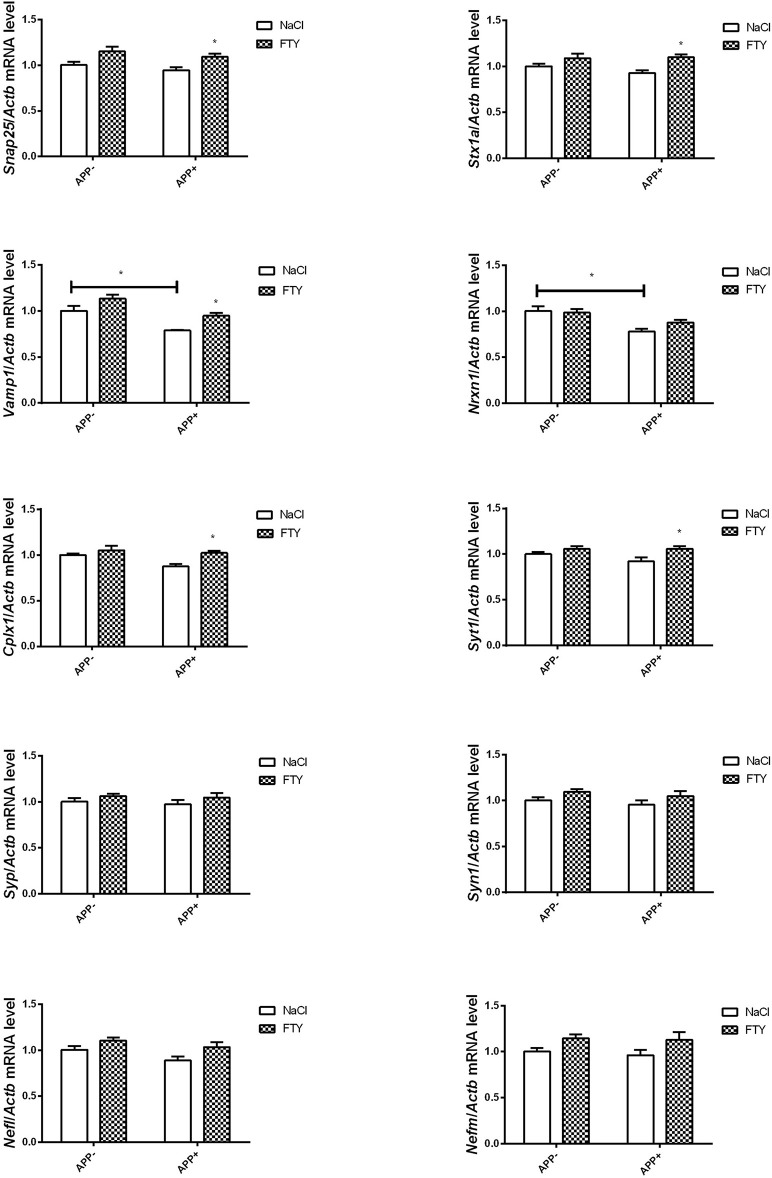
Levels of mRNAs coding for synaptic proteins in 12-month-old cortex: effect of AβPP and FTY720. Levels of mRNAs measured with real-time PCR in the cerebral cortex of 12-month-old (old adult) mice as described in Materials and methods. mRNA levels in AβPP-expressing mice were compared with control animals that did not inherit the transgene. Effect of FTY720 administration was assessed against vehicle-treated animals in each group. *Snap25*, synaptosomal-associated protein, 25kDa; *Stx1a*, syntaxin 1A; *Vamp1*, vesicle-associated membrane protein 1; *Nrxn1*, neurexin 1; *Cplx1*, complexin 1; *Syt1*, synaptotagmin 1; *Syp*, synaptophysin; *Syn1*, synapsin 1; *Nefl*, neurofilament light; *Nefm*, neurofilament medium; APP^−^, animals without V717I AβPP transgene; APP^+^, mice expressing V717I AβPP transgene. ^*^p < 0.05 vs. corresponding controls, two-way ANOVA followed by Tukey's *post-hoc* test (horizontal bars: effect of AβPP; significances marked over FTY720 values: effect of FTY720 treatment within each animal group). *n* = 3–6, measured in triplicate (*n* = 6–9 for *Cplx1, Snap25, Syn1*, and *Nefl*) ± SEM.

Twelve-month-old mice expressing V717I AβPP demonstrated reduced expression of numerous genes coding for synaptic proteins. *Vamp1* mRNA was again significantly reduced in AβPP-expressing brain cortex compared with controls that did not inherit the transgene (Figure 3). FTY720 treatment returned its expression to near control levels. *Nrxn1* mRNA was also reduced by AβPP expression, but FTY720 had no effect on it. No changes were observed in 12-month-old brain cortex in the mRNA levels of *Stx1a, Cplx1, Syt1, Syp, Syn1*, or *Snap25* in response to AβPP (Figure 3). However, FTY720 treatment increased *Cplx1, Snap25, Stx1a*, and *Syt1* in the transgenic animals. *Nefl* and *Nefm* remained unchanged (Figure 3).

**Figure 4:**
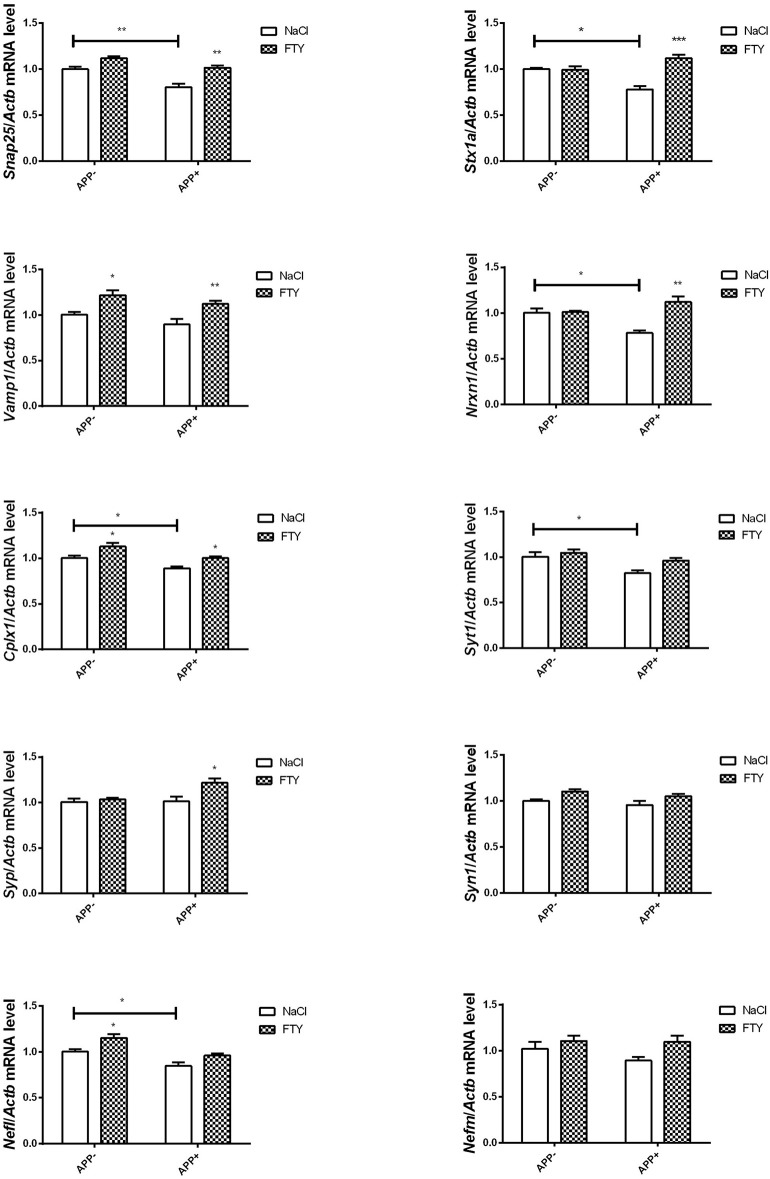
Levels of mRNAs coding for synaptic proteins in 12-month-old hippocampus: effect of AβPP and FTY720. Levels of mRNAs measured with real-time PCR in the hippocampus of 12-month-old (old adult) mice as described in Materials and methods. mRNA levels in AβPP-expressing mice were compared with control animals that did not inherit the transgene. Effect of FTY720 administration was assessed against vehicle-treated animals in each group. *Snap25*, synaptosomal-associated protein, 25kDa; *Stx1a*, syntaxin 1A; *Vamp1*, vesicle-associated membrane protein 1; *Nrxn1*, neurexin 1; *Cplx1*, complexin 1; *Syt1*, synaptotagmin 1; *Syp*, synaptophysin; *Syn1*, synapsin 1; *Nefl*, neurofilament light; *Nefm*, neurofilament medium; APP^−^, animals without V717I AβPP transgene; APP^+^, mice expressing V717I AβPP transgene. ^*^p < 0.05; ^**^p < 0.01; ^***^p < 0.001 vs. corresponding controls, two-way ANOVA followed by Tukey's *post-hoc* test (horizontal bars: effect of AβPP; significances marked over FTY720 values: effect of FTY720 treatment within each animal group). *n* = 3–6, measured in triplicate (*n* = 7–14 for *Cplx1, Syp, Syn1, Vamp1*, and *Nefl*) ± SEM.

Also, at 12 months, hippocampal mRNA levels were significantly lower in AβPP-expressing animals compared with non-transgenic controls: *Snap25, Stx1a, Nrxn1*, and *Cplx1*. FTY720 reversed these changes. *Syt1* mRNA was also reduced in AβPP-expressing hippocampus, but FTY720 did not change it significantly. *Vamp1* and *Syp* remained unaltered by AβPP expression, but both responded positively to FTY720. We also observed that *Nefl* mRNA dropped significantly in AβPP animals, while *Syn1* and *Nefm* remained unchanged (Figure 4).

**Figure 5:**
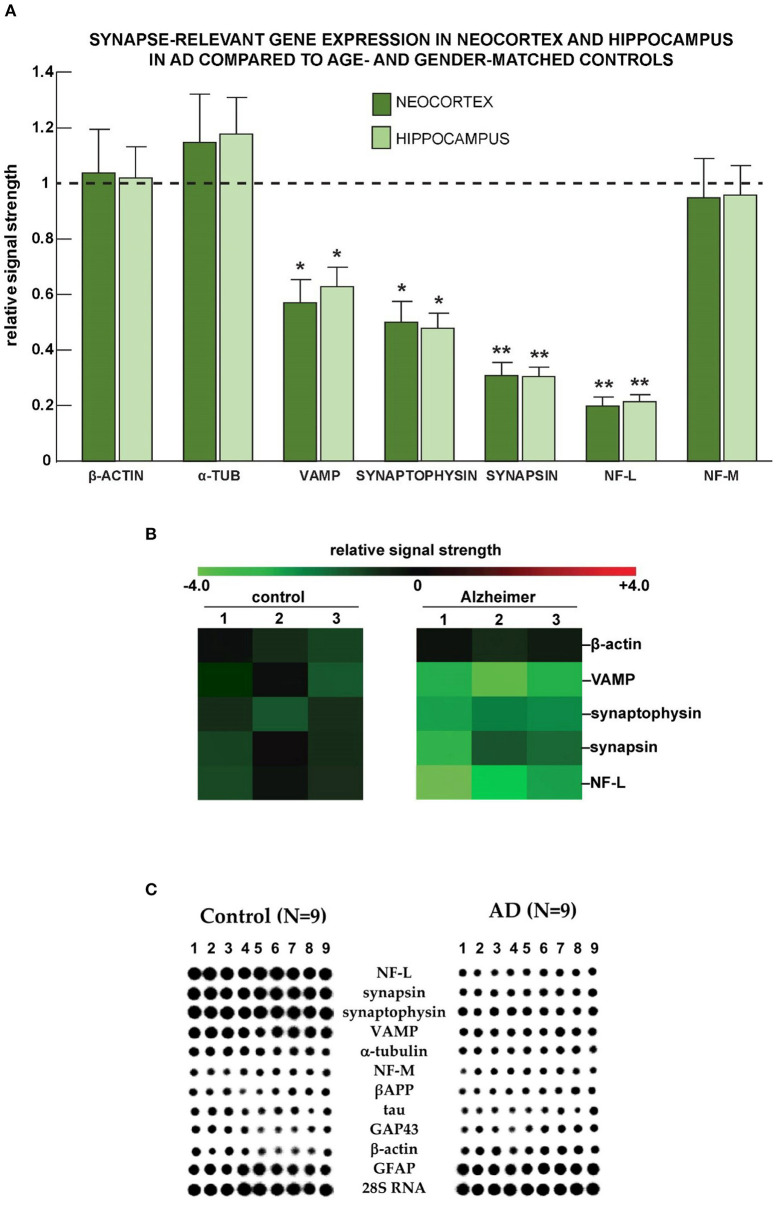
Selective down-regulation of the expression of genes coding for synaptic proteins in AD. **(A)** Neocortical and hippocampal down-regulation of VAMP, synaptophysin, synapsin, and the neuron-specific neurofilament light (NF-L) chain messenger RNA in Alzheimer's disease vs. age-, gender-, and post mortem interval-matched controls using the β-actin cytoskeletal as an internal “housekeeping gene” and expression control. Importantly, the low abundance of the neuron-specific NF-L mRNA cannot be adequately accounted for by a non-specific effect of brain damage, neuron cell loss, or loss of neurons with neurofibrillary degeneration (McLachlan et al., 1988; Lukiw et al., 2018). The AD group (*N* = 3) had a mean age of 76.1 ± 11.4 years and a mean post mortem (PMI; death to brain-freezing period) of ~3.4 h and the control group (*N* = 3) had a mean age of 75.5 ± 12.7 years and a mean PMI of ~3.5 h; all brain samples were from female donors; there was no significant difference in the mean age, gender, PMI, or yield of total RNA between the AD and control groups. Total RNA was extracted and probed for α-tubulin, VAMP, synaptophysin, synapsin, the neurofilament light and medium neurofilaments, and quantified based on unchanging β-actin control levels in the same sample using methods explained in detail in previously published study from our laboratories (McLachlan et al., 1988; Lukiw et al., 2008, 2018); deficits in synapse-relevant gene expression were consistently found to be NF-L < synapsin < synaptophysin < VAMP; these observations are also consistent with synaptic signaling deficits in AD brain compared to three unchanging biomarkers: β-actin, α-tubulin, and the NF-M chain protein; ^*^p < 0.05; ^**^p < 0.01 (ANOVA). **(B)** Down-regulation of mRNAs coding for vesicle-associated membrane protein (VAMP), synaptophysin (SYP), synapsin, and NF-L in Alzheimer's disease vs. age- gender- and post-mortem interval-matched controls using the β-actin cytoskeletal as a “housekeeping gene” and expression control using DNA array analysis (McLachlan et al., 1988; Lukiw et al., 2008, 2018); the numbers 1–3 along the top represent results from *N* = 3 control and *N* = 3 Alzheimer cases; again, all were age-matched from female donors; there was no significant difference in the mean age, PMI, or yield of total RNA between the AD and control groups. **(C)** Selective deficits for synapse-relevant gene expression were also observed in AD temporal lobe neocortex compared with age- and gender-matched controls using Northern dot blot analysis. Deficits in gene expression for synaptic components in control (*N* = 9) vs. AD (*N* = 9) female superior temporal lobe (Brodmann A22) neocortices using quantitative Northern dot blots for signal quantitation; the PMI for all tissues was ~4 h or less; a 288-gene matrix was generated using a Biomek 2000 robot (Beckman-Coulter Life Sciences, Indianapolis, IN, United States) at a Northern dot blot density of about 30 genes/cm^2^; in **(C)**, each vertical column represents the Northern dot blot signature of one control or one AD brain, and each horizontal row represents the hybridization signals from one particular synaptic-relevant probe; signal intensity of each dot in the dot blot is proportional to the abundance of that specific mRNA in the sample; note significant deficits in signal strength (signal intensity of the dot blot) for NF-L, synapsin, synaptophysin, and vesicle-associated membrane protein (VAMP) and negligible changes between control and AD for α-tubulin, NF-M, the amyloid precursor protein (APP), tau, the 43 kDa growth associated protein GAP43 (neuromodulin), the cytoskeletal protein β-actin, glial fibrillary acidic protein (GFAP), and the large ribosomal subunit 28S RNA. In these DNA array studies, the control group (*N* = 9) had a mean age of 77.5 ± 13.5 years, and the AD group (*N* = 9) had a mean age of 78.5 ± 11.8 years; all brain samples were from female donors; again, there was no significant difference in the mean age, gender, PMI, or yield of total RNA between the AD and control groups. Experimental values in all of the Figures are expressed as mean ± standard deviation of that mean. This figure represents updated and additional new information for NF-L gene expression data adapted from earlier studies on specific cytoskeletal gene abundance in control and AD neocortex (McLachlan et al., 1988; Lukiw et al., 1990).

Analysis of data from nine human AD samples (Figure 5) indicated a reduced expression of *VAMP*, synaptophysin, synapsin, and *NF-L* in comparison to 15 healthy controls. The changes were significant in both neocortical and hippocampal materials. Similar results were obtained using Northern dot blot array methodologies.

## Discussion

Our previous analysis (Jeśko et al., 2019b, 2020) has shown that the reaction of mouse brain to the expression of AβPP (V717I) transgene is highly different at various ages. The presence of AβPP in adult (3-month-old) animals was associated with elevated ceramide turnover (higher expression of both ceramide-synthesizing and -degrading enzymes of the salvage pathway of sphingolipid metabolism), but 12-month-old transgenic mice only displayed a reduction in the expression of ceramide-utilizing enzymes in the sphingomyelinase pathway. The effects of such changes may include the well-documented intensification of pro-apoptotic signaling. Literature points to an early rise in the predominantly pro-apoptotic ceramide and disturbed production of S1P, which, in most situations, supports cell survival, in human AD brains. Disturbances in ceramide/sphingolipid metabolism appear very early (leading to the proposal of *pre-MCI* phase that would precede the mild cognitive impairment), correlate with the severity of neurodegeneration, and, strikingly, diminish in later disease stages (Katsel et al., 2007; Han, 2010; Couttas et al., 2014).

Altered sphingolipid signaling may also heavily affect neuronal phenotype, as sphingolipids regulate glutamate secretion, the expression of glutamate receptor subunits, and probably the shape and structure of neurites and synapses (Riganti et al., 2016; Joshi et al., 2017). Synapse loss is one of the early features of AD and correlates with the ongoing cognitive deterioration (Scheff et al., 2006; Ferreira et al., 2015). Deregulation and eventual degeneration of synaptic connections is largely dependent on Aβ levels (Forner et al., 2019; Sharda et al., 2020). Disruption of lipid rafts, which are sphingolipid-enriched microdomains in the neuronal membrane, is a known trigger of synapse loss (Hering et al., 2003). Inhibition of ceramide synthesis, or exogenous addition of sphingosine, causes axon growth blockade and axon retraction in cultured neurons (Campenot et al., 1991; Harel and Futerman, 1993). Inhibition of ceramide synthase also disturbs dendrite formation in cultured neurons (Furuya et al., 1995). Manipulation of ceramide metabolism alters numerous neuronal signaling systems, such as NMDA, AMPA (α-amino-3-hydroxy-5-methyl-4-isoxazolepropionic acid), and acetylcholine receptors, reviewed in Olsen and Færgeman (2017). Metabolism of AβPP and production of Aβ are also highly dependent on the rafts (Czubowicz et al., 2019). The synaptic targets that bind Aβ and may mediate synapse loss could include some neurexin and neuroligin isoforms, glutamate, adrenergic, and nicotinic receptors, calcium channels, or GM1 ganglioside (Ferreira et al., 2015; Brito-Moreira et al., 2017). However, Aβ might also deregulate synaptic homeostasis through alteration of the role of sphingolipids in gene regulation (Jeśko et al., 2019b, 2020), which is mostly realized through cell surface S1P receptors (S1PR1-5), G proteins, and the PI3K (phosphoinositide 3-kinase)–Akt pathway (Jeśko et al., 2019a). PI3K/Akt–dependent transcription factors include AP-1 and NF-κB. Interestingly, promoter of the human SNAP25 synaptic protein gene contains sites binding sphingolipid-regulated transcription factors AP-1 and Sp1 (specificity protein 1) (Cai et al., 2008; Zou et al., 2011; Hsu et al., 2015). The potential of Aβ to cause synaptic disruption prompted us to analyze the levels of mRNA coding for selected synaptic proteins in the AβPP (V717I) transgenic mouse model.

The results showed decreased expression of several crucial genes coding SNARE proteins and their interacting partners in (V717I) AβPP transgenic mice. At the age of 3 months, the changes were limited to reduced *Vamp1* mRNA (both in the cerebral cortex and hippocampus) and *Syt1* (only in the hippocampus), (Figures 1, 2). These changes might signify an already ongoing disruption of synaptic structure. Administration of the potentially neuroprotective S1P receptor modulator FTY720 has not changed the abnormal expression of *Syt1* or *Vamp1* in 3-month-old AβPP transgenic mice.

In contrast to the young adults, we observed numerous gene expression changes in 12-month-old AβPP-expressing animals. *Vamp1* and *Nrxn1* mRNAs were reduced in the cerebral cortex, while other measured mRNAs (such as *Cplx1, Snap25, Syp*, and *Stx1a*) remained at the same level as in non-AβPP controls (Figure 3). *Nrxn1* mRNA was reduced in both investigated 12-month-old brain parts. FTY720 reversed the decrease in *Vamp1* but not *Nrxn1* mRNAs; and the compound also had a positive influence on *Cplx1, Snap25, Stx1a*, and *Syt1* in the 12-month-old animals (Figure 3).

Opposite to the cerebral cortex, the 12-month-old hippocampus displayed a nearly uniform reduction in *Cplx1, Snap25, Stx1a, Nrxn1, Syt1*, and *Nefl* but not *Vamp1* mRNA, which seems to reflect unique sensitivity of the brain part to the gradually accumulating effects of Aβ/AβPP. The reduction in *Snap25, Syt1* as well as *Vamp1* hippocampal gene expression is consistent with previously observed changes in the human AD hippocampus (Berchtold et al., 2013). Importantly, reduction in *Cplx1, Snap25, Stx1a*, and *Nrxn1* could be reversed by FTY720, which also increased the levels of *Vamp1* and *Syp*.

We next evaluated the expression of selected synaptic protein-coding genes in samples from high quality human brain tissues with post mortem intervals (PMIs) of 2.4 h or less (Figure 5). Interestingly, the patterns of AD-linked changes in the human hippocampus and neocortex were very similar to each other, but the set of genes affected showed a noticeable similarity to the mouse model. We observed reduced expression of *VAMP, SYP, SYN*, and *NF-L* (*NEFL*) mRNAs, in both brain regions.

VAMP1, STX1A, and SNAP25 belong to the SNARE complex critically implicated in vesicular secretion of neurotransmitters, in the membrane insertion of receptor proteins, and also in synaptic plasticity, axon guidance, or nerve regeneration (Ulloa et al., 2018; Madrigal et al., 2019). Several lines of evidence suggest the possible engagement of SNARE proteins in AD, although data are rather limited: polymorphisms in *VAMP1* and *STX* genes associate with AD; VAMP1 can modulate Aβ secretion; while VAMP2 levels are disturbed in AD (Sevlever et al., 2015; Vallortigara et al., 2016; Costa et al., 2019). The SNARE trimer binds a multitude of other proteins, such as regulators (synaptotagmin–SYT, complexin–CPLX), and also α-synuclein (Almandoz-Gil et al., 2018; Alford et al., 2019; Hawk et al., 2019; Karmakar et al., 2019). SYT1 is engaged in Ca^2+^ sensing and coupling of calcium signal to neurotransmitter release; genetic *SYT* ablation can lead to disturbed synaptic vesicle exocytosis, such as uncontrolled (spontaneous) release (Volynski and Krishnakumar, 2018). SYT1, -2, -7, and -9 bind AβPP; SYT1 probably supports AβPP processing by β-secretase (Gautam et al., 2015; Barthet et al., 2018). SYT1 also interacts with presenilin 1, and experimental disruption of their interaction increases the proportion of the highly neurotoxic Aβ_42_ (Zoltowska et al., 2017). Moreover, SYT1 levels and SYT1–presenilin 1 binding are disturbed in the brain of humans with AD (Zoltowska et al., 2017).

Neurexins (NRXN) are single-pass transmembrane proteins located predominantly in the presynaptic part; they bind post-synaptic neuroligins (NLGN), neurexophilins, and dystroglycan systems ensuring structural properties of neuronal connections. Neurexins also influence differentiation and synaptic vesicle production. Their sensitivity to calcium allows for direct regulation of NLGN–NRXN binding by neuron activity (Sindi et al., 2014). Neurexin mutations may predispose to AD (Sindi et al., 2014). Aβ impairs the expression of *NRXN1*β and can bind NRXN2A (Brito-Moreira et al., 2017; Naito et al., 2017). Interactions of NRXN with Aβ/secretases can lead to, e.g., accumulation of NRXN fragments in the extracellular space, membranes, and pre-synaptic cytoplasm, and finally to altered efficiency of synaptic transmission [discussed in Sindi et al. (2014)] but also to structural disruption and loss of synapses (Brito-Moreira et al., 2017; Naito et al., 2017). The reduction of *Nrxn1* and *Vamp1* expression we observed might be a part of advancing synaptic deterioration. However, the known association of AD risk with *VAMP1* polymorphisms that cause increased transcription, and the positive influence of VAMP1 on Aβ secretion (Sevlever et al., 2015) suggest an ambiguous role for the protein. We cannot rule out a neuroprotective outcome of *Vamp1* reduction, and some potentially homeostatic reactions might occur even relatively late in the disease progression.

Synaptophysin (SYP) is a cholesterol-binding membrane protein that transiently interacts with VAMP in an activity-dependent manner (Hübner et al., 2002; Khvotchev and Südhof, 2004). The significance of SYP for synaptic function/maintenance is unclear because of the lack of gross gene ablation-induced phenotypes. However, reduced synaptophysin has long been noted in aging and in a disease-specific, spatially-restricted manner in early stages of AD and some other neurodegeneration types (Masliah et al., 1989; Lippa, 2004; Martin et al., 2014). Reduced SYP also correlates with AD severity (Sze et al., 1997; Heffernan et al., 1998). Loss of SYP staining is observed in neurons in the vicinity of Aβ oligomer deposits (Ishibashi et al., 2006); interaction of SYP with internalized Aβ and Aβ-induced preferential SYP nitration have been suggested to mediate synaptic disturbances observed in AD (Tran et al., 2003; Russell et al., 2012). Reduction of *Syp* expression was also observed in neurons containing neurofibrillary tangles (Callahan et al., 1999).

Synapsins (SYN) are highly abundant, neuron-specific pre-synaptic vesicle proteins engaged in synaptogenesis, regulation of vesicle storage, fusion, and resulting neurotransmitter release (Song and Augustine, 2015). SYN disturbances are noted in multiple neurodegenerative/psychiatric disorders, including AD (Qin et al., 2004; Song and Augustine, 2015). Specifically, loss of SYN1 has been previously observed in some layers of CA1 and dentate gyrus of patients with AD hippocampus (Qin et al., 2004). Moreover, a reduction in *Syn1* mRNA levels was observed in the CA3 layer of the hippocampus of rats with sporadic AD (Bolognin et al., 2012). Similar results were also reported by Berchtold et al. who observed structure-specific downregulation of *SYN1* mRNA levels in the hippocampus of patients with AD. Interestingly, cortical *SYN1* mRNA reduction was observed in the aged control group (69–99 vs. 20–50 years) (Berchtold et al., 2013).

The results also confirm reduced expression of *NEFL* (NF-L), which encodes the 68 kDa neurofilament protein. NF-L is a neuron-specific intermediate filament being a critical scaffolding component of neurite extensions, the primary regulator of axon diameter, overall neuronal cytoarchitecture, neuron shape and morphology, and an integral component of synaptic complexes (Palermo et al., 2020). Down-regulated NF-L within degenerating neocortical neurons, such as those seen in AD, correlates well with the neuronal atrophy and deterioration widely observed in progressive neurological degeneration (Berchtold et al., 2013). Diminished brain levels of NF-L have been reported by several independent laboratories in AD and in transgenic murine models for AD (TgAD) irrespective of reduced neuron count (Lukiw et al., 1990, 2018; Loeffler et al., 2020). NF-L may be a diagnostic biomarker of brain atrophy and disease progression in multiple nervous system disorders (Gaetani et al., 2019; Antonell et al., 2020).

The difference between human and rodent samples could stem from several sources. First, although the (V717I) AβPP-expressing mice relatively successfully recreate a spectrum of AD pathology aspects, they still share the obvious limitations of all rodent AD models. Second, human post mortem samples are collected at much more advanced stages of widespread, massive neurodegeneration, in contrast to the mild degenerative changes observed in 12-month-old (V717I) AβPP mouse brains. Moreover, some differences in the spatial distribution of sensitivity to Aβ neurotoxicity between the human and mouse brains might also modify the outcome.

**Figure 6:**
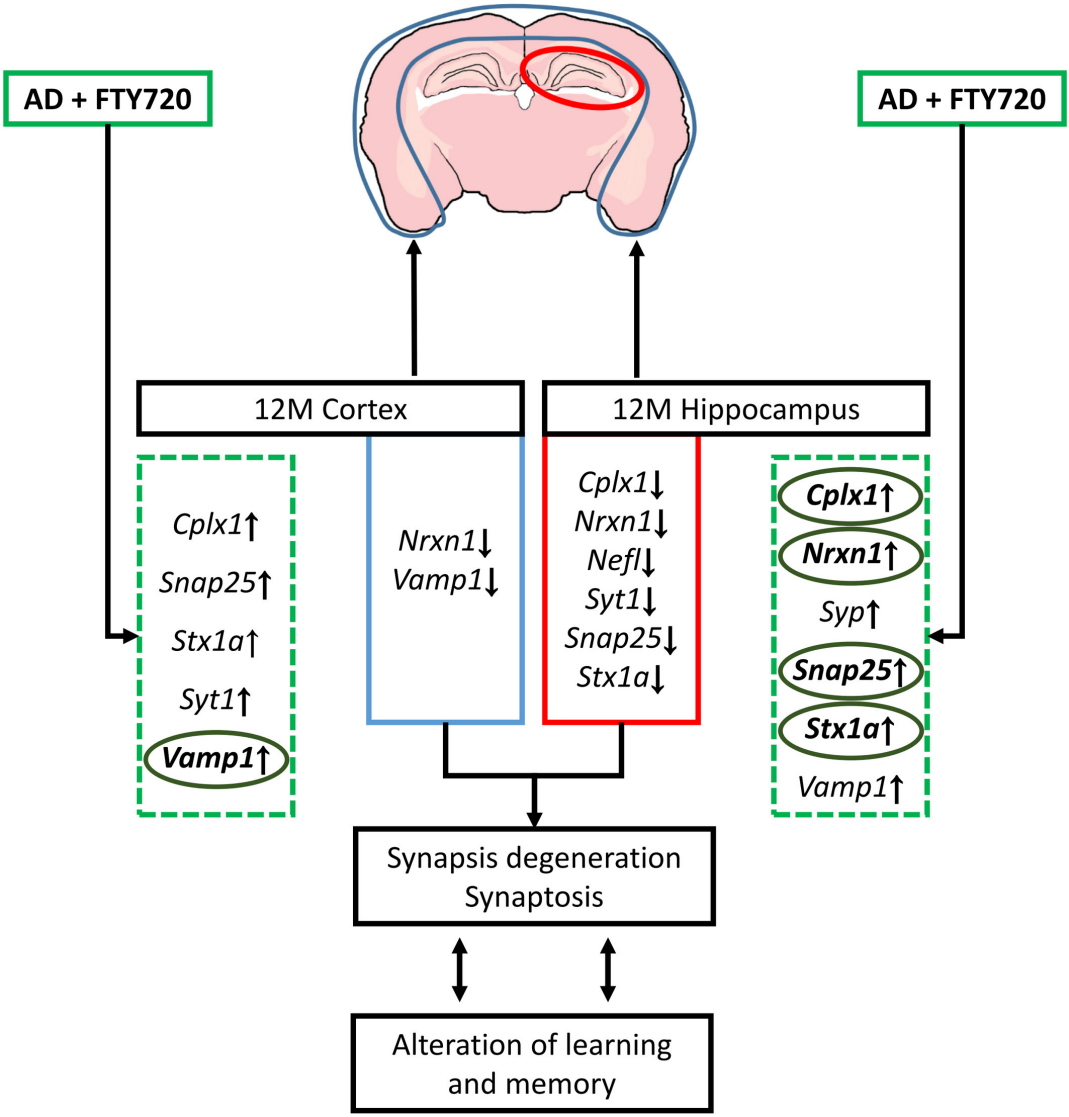
Schematic representation of changes in genes coding synaptic proteins and their possible consequences observed in 12-month (12 M) FVB/AβPP (V717I) mice cortex and hippocampus. The effect of fingolimod (FTY720, marked by green dashed square) on genes that expression was altered in AD mice brain was bolded and marked with circles (ellipses). *Snap25*, synaptosomal-associated protein, 25kDa; *Stx1a*, syntaxin 1A; *Vamp1*, vesicle-associated membrane protein 1; *Nrxn1*, neurexin 1; *Cplx1*, complexin 1; *Syt1*, synaptotagmin 1; *Syp*, synaptophysin; *Nefl*, neurofilament light.

The results, therefore, show that the impact of Aβ/AβPP on critical synaptic components is highly dependent on age/disease stage and is brain part specific, with the highest number of changes occurring in the 12-month-old hippocampi (all changes that were observed in 12-month-old transgenic mice were presented in Figure 6). FTY720 is capable of reversing many of Aβ/AβPP-induced changes in the expression of synaptic proteins, suggesting its value as a research tool and possibly a repurposed drug. Although the mouse model obviously examines the disease at a much earlier stage (and at a much younger age) than observed in human tissues, we noted several striking similarities in the obtained results. Therefore, we can suggest that the sensitivity of several key synaptic components to FTY720 administration may also be present in the human nervous system. Synaptic deterioration is a relatively early stage of degeneration in AD, but its biochemical manifestations appear to persist to the end stage.

The pleiotropic activities of FTY720 require further attention to the potential mechanism of its restorative action. One plausible explanation is the ability of S1P receptors to modulate transcription factors, such as AP-1 or NF-kappa B, therefore relatively directly and specifically affecting the transcription of multiple genes (including feedback regulation of sphingolipid metabolic enzymes) (Jeśko et al., 2019a,b; Jeśko et al., 2020). S1P and its receptors also have a complex influence on PI3 kinase/Akt and their signaling target mTOR, potentially influencing gene transcription and translation through ribosomal protein S6 kinases, eukaryotic translation initiation factor 4E-binding proteins, and FOXO transcription factors (Jeśko et al., 2019a). Both sphingosine kinase 2 and its product S1P can modulate histone deacetylases (Jeśko et al., 2019a). More general influences of FTY720 on AβPP metabolism and inflammation may add further layers of complexity. FTY720, acting as a ligand, can cause regulatory internalization of S1PR protein, effectively inhibiting S1PR signaling in lymphocytes and blocking their egress from secondary lymphoid organs. This effect is exploited in the therapy of relapsing remitting multiple sclerosis (Sica et al., 2019). FTY720 also reduces the numbers of activated microglia and astrocytes in the brain of rodent AD models, normalizing cytokines, synaptic morphology, plasticity, and learning performance (Hemmati et al., 2013; Aytan et al., 2016). Anti-inflammatory effects of FTY720 have also been shown in other diseases, such as amyotrophic lateral sclerosis (ALS), Parkinson's disease (PD), Huntington's disease (HD), neuronal ceroid lipofuscinoses, and neonatal hyperoxia (Bascuñana et al., 2020). Although feedback S1PR internalization upon ligation has been considered the main mechanism of the anti-inflammatory action of FTY720, the atypical dose-response characteristics suggest that agonistic action on S1PRs may also be important in this case, possibly involving two different mechanisms depending on compound concentration (Aytan et al., 2016). Moreover, FTY720 might be most effective in concentrations that reduce microglia and astrocyte activation but do not affect peripheral lymphocytes (Carreras et al., 2019).

Much study has also been conducted on the effects of FTY720 on amyloid beta accumulation. FTY720 can decrease the accumulation of soluble and plaque Aβ, probably through an increased phagocytic capacity of astrocytes and reduced microgliosis (McManus et al., 2017; Kartalou et al., 2020). Takasugi observed that reduction of Aβ load by FTY720 and change in the proportions between Aβ40 and Aβ42 may occur in the presence of Gi inhibitor suramin, therefore likely independently of the currently known S1PR-dependent signaling pathways, possibly through direct binding of FTY720 to γ-secretase or AβPP (FTY720 decreased the γ-secretase mediated cleavage of AβPP) (Takasugi et al., 2013).

The gradual evolution of the Aβ/AβPP-associated changes in synaptic composition we observed in aging AβPP transgenic mice occurs along with the known Aβ-induced disruption of synaptic protein-protein interactions (Marsh and Alifragis, 2018). This phenomenon suggests the necessity of wide, in-depth characterization of the feasibility of Aβ-induced synaptic changes as a potential druggable target in AD. Irrespective of the mentioned cautions, the observed effects of FTY720 administration on synaptic protein expression suggest restorative potential, in accordance with the currently prevailing view on the action of FTY720 in AD and other neurodegenerative disorders.

## Data Availability Statement

The original contributions presented in the study are included in the article/Supplementary Material, further inquiries can be directed to the corresponding author/s.

## Ethics Statement

The studies involving human participants were reviewed and approved by the ethical use of postmortem human brain tissues and their analyses were also carried out in strict accordance with the Institutional Biosafety Committee and the Institutional Review Board Committee (IBC/IRBC) ethical guidelines IBC#18059 and IRBC#6774 at the LSU Health Sciences Center, New Orleans LA 70112 USA. Project identification codes: NIA AG18031 and NIA AG038834. Written informed consent for participation was not required for this study in accordance with the national legislation and the institutional requirements. The animal study was reviewed and approved by the IV Local Ethics Committee for Animal Experimentation in Warsaw and the Ministry of Environment (approval no. 67/2015 from 2nd July 2016 and no. 139 from 22nd August 2016, respectively) and were carried out in accordance with the EC Council Directive of November 24, 1986 (86/609/EEC) following the ARRIVE guidelines, the NIH Guide for the Care and Use of Laboratory Animals, and the Guidelines for the Use of Animals in Neuroscience Research by the Society for Neuroscience.

## Author Contributions

RS and WL: conceptualization and formal analysis. HJ, IW, PW, RS, and WL: methodology and investigation. RS: validation, resources, supervision, project administration, and funding acquisition. RS and PW: data curation. HJ: writing – original draft preparation. HJ and RS: writing – review and editing. IW and PW: visualization. All authors contributed to the article and approved the submitted version.

## Conflict of Interest

The authors declare that the research was conducted in the absence of any commercial or financial relationships that could be construed as a potential conflict of interest.

## Supplementary Material

Supplementary Figure 1 Levels of mRNA coding for synaptic proteins in 3-month-old mouse cortex: effect of AβPP expression and FTY720 administration. Levels of mRNAs measured with real-time PCR in the cerebral cortex of 3-month-old (adult) mice as described in Materials and methods. Results from AβPP-expressing mice were compared with those from control animals that did not inherit the transgene. Effect of FTY720 administration was assessed against vehicle-treated animals of the same group. *Snap25*, synaptosomal-associated protein, 25kDa; *Stx1a*, syntaxin 1A; *Nrxn1*, neurexin 1; *Cplx1*, complexin 1; Syt, synaptotagmin 1; *Syp*, synaptophysin; *Syn1*, synapsin 1; *Nefl*, neurofilament light; *Nefm*, neurofilament medium; APP^−^, animals without V717I AβPP transgene; APP^+^, mice expressing V717I AβPP transgene. *n* = 3–4 (for *Nrxn1, Cplx1, Syt1*, and *Syp*), *n* = 6–8 (for *Snap25, Stx1a, Syn1, Nefl*, and *Nefm*) measured in triplicate.

Supplementary Figure 2 Levels of mRNA coding for synaptic proteins in 3-month-old mouse hippocampus: effect of AβPP expression and FTY720 administration. Levels of mRNAs measured with real-time PCR in the hippocampus of 3-month-old (adult) mice as described in Materials and methods. Results from AβPP-expressing mice were compared with those from control animals that did not inherit the transgene. Effect of FTY720 administration was assessed against vehicle-treated animals of the same group. *Snap25*, synaptosomal-associated protein, 25kDa; *Stx1a*, syntaxin 1A; *Nrxn1*, neurexin 1; *Cplx1*, complexin 1; *Syp*, synaptophysin; *Syn1*, synapsin 1; *Nefl*, neurofilament light; *Nefm*, neurofilament medium; APP^−^, animals without V717I AβPP transgene; APP^+^, mice expressing V717I AβPP transgene. *n* = 3–4 measured in triplicate (for *Snap25, Nrxn1, Syp, Syn1, Nefl*, and *Nefm*) (*n* = 6–8 for *Stx1a* and *Cplx1*).

## Abbreviations

Aβ: amyloid β
AβPP: Aβ precursor protein
ACTB: actin beta
AD: Alzheimer's disease
CNS: central nervous system
CPLX1: complexin 1
FTY720: fingolimod/Gilenya™
GAP43: Growth-associated protein 43/neuromodulin
GFAP: glial fibrillary acidic protein
NF-L: neurofilament light chain
NF-M: neurofilament medium chain
NRXN1: neurexin 1
S1P: sphingosine-1-phosphate
S1PR: S1P receptor
SNAP25: synaptosomal-associated protein 25kDa
SNARE: soluble N-ethylmaleimide sensitive fusion attachment protein receptor
Sp1: specificity protein 1
STX1A: syntaxin 1A
SYN1: synapsin 1
SYT1: synaptotagmin 1
SYP: synaptophysin
t-SNARE: target membrane SNARE protein
VAMP1: vesicle-associated membrane protein 1
v-SNARE: vesicular side SNARE protein.
