# Supplementary figures and images for: Age-Related Transcriptional Deregulation of Genes Coding Synaptic Proteins in Alzheimer's Disease Murine Model: Potential Neuroprotective Effect of Fingolimod
Source: Front Mol Neurosci. 2021 Jul 9;14:660104. doi: 10.3389/fnmol.2021.660104 (PMC8299068; doi:10.3389/fnmol.2021.660104)

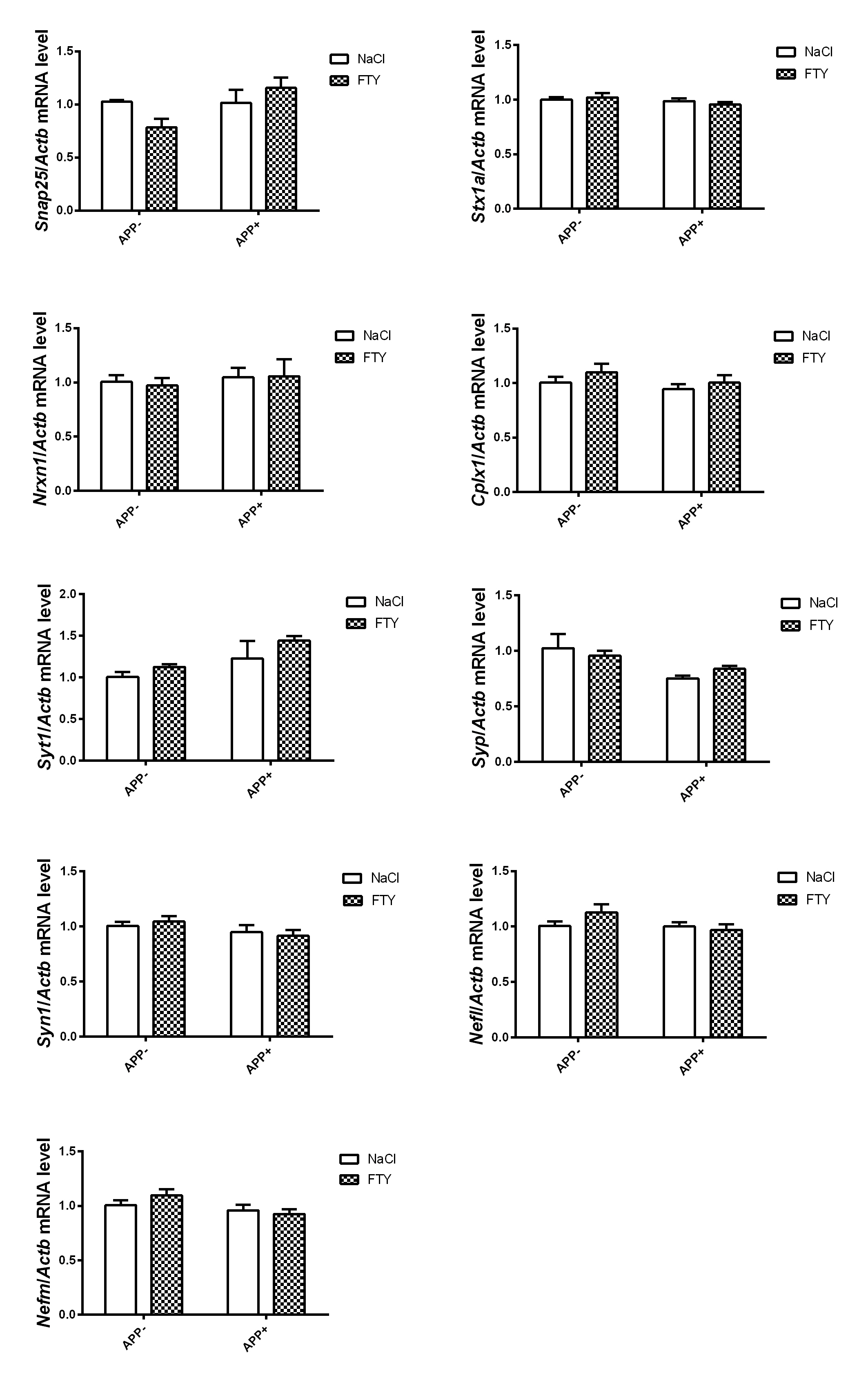

Supplement: Supplementary Figure 1 — Levels of mRNA coding for synaptic proteins in 3-month-old mouse cortex: effect of AβPP expression and FTY720 administration. Levels of mRNAs measured with real-time PCR in the cerebral cortex of 3-month-old (adult) mice as described in Materials and methods. Results from AβPP-expressing mice were compared with those from control animals that did not inherit the transgene. Effect of FTY720 administration was assessed against vehicle-treated animals of the same group. Snap25, synaptosomal-associated protein, 25kDa; Stx1a, syntaxin 1A; Nrxn1, neurexin 1; Cplx1, complexin 1; Syt, synaptotagmin 1; Syp, synaptophysin; Syn1, synapsin 1; Nefl, neurofilament light; Nefm, neurofilament medium; APP−, animals without V717I AβPP transgene; APP+, mice expressing V717I AβPP transgene. n = 3–4 (for Nrxn1, Cplx1, Syt1, and Syp), n = 6–8 (for Snap25, Stx1a, Syn1, Nefl, and Nefm) measured in triplicate. [file Image_1.JPEG]

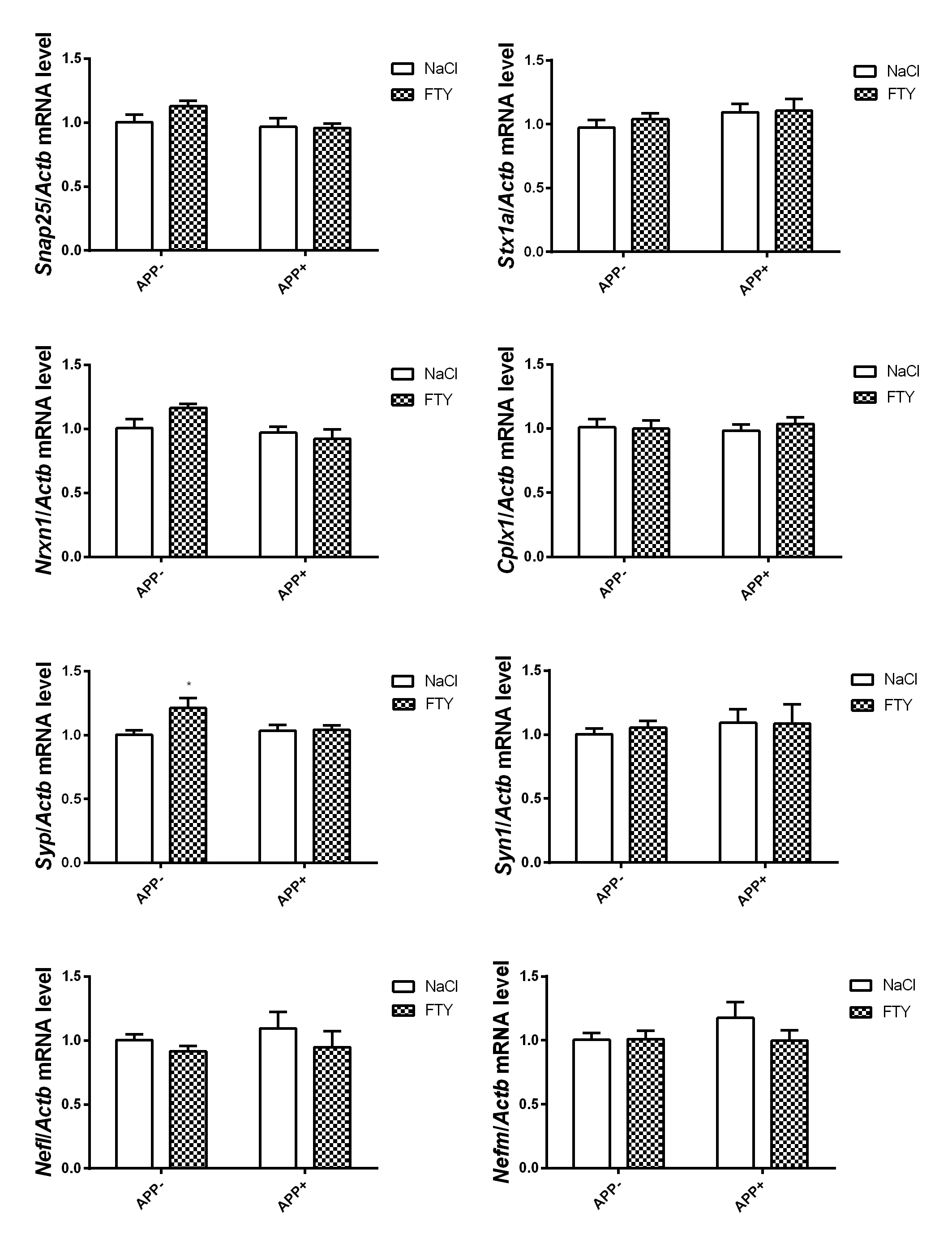

Supplement: Supplementary Figure 2 — Levels of mRNA coding for synaptic proteins in 3-month-old mouse hippocampus: effect of AβPP expression and FTY720 administration. Levels of mRNAs measured with real-time PCR in the hippocampus of 3-month-old (adult) mice as described in Materials and methods. Results from AβPP-expressing mice were compared with those from control animals that did not inherit the transgene. Effect of FTY720 administration was assessed against vehicle-treated animals of the same group. Snap25, synaptosomal-associated protein, 25kDa; Stx1a, syntaxin 1A; Nrxn1, neurexin 1; Cplx1, complexin 1; Syp, synaptophysin; Syn1, synapsin 1; Nefl, neurofilament light; Nefm, neurofilament medium; APP−, animals without V717I AβPP transgene; APP+, mice expressing V717I AβPP transgene. n = 3–4 measured in triplicate (for Snap25, Nrxn1, Syp, Syn1, Nefl, and Nefm) (n = 6–8 for Stx1a and Cplx1). [file Image_2.JPEG]
